# Effectiveness of Transcutaneous and Percutaneous Electrical Nerve Stimulation as Adjunct Therapies in Patients After Anterior Cruciate Ligament Reconstruction: Study Protocol for a Randomized Controlled Trial

**DOI:** 10.3390/jcm15030989

**Published:** 2026-01-26

**Authors:** Luis Blanco-López, Iván Nácher-Moltò, Juan Luis Sánchez-González, Daniel Casado-Gómez, Adrián Cases-Sebastià, Javier Reina-Abellán

**Affiliations:** 1 Health Sciences PhD Program, Universidad Católica San Antonio de Murcia, Campus de los Jerónimos, 135, 30107 Murcia, Spain; lblanco5@alu.ucam.edu (L.B.-L.); acases5@alu.ucam.edu (A.C.-S.); 2 Clínica CEMTRO, 28035 Madrid, Spain; 3 Physiotherapy Department, Universidad CEU Cardenal Herrera, Campus Alfara del Patriarca, 46115 Valencia, Spain; 4 Department of Medicine, Universidad de Salamanca, Campus Miguel de Unamuno, 37007 Salamanca, Spain; juanluissanchez@usal.es; 5 Institute of Biomedical Research of Salamanca (IBSAL), 37007 Salamanca, Spain; 6 Physiotherapy Department, Universidad Católica San Antonio de Murcia, Campus de los Jerónimos, 135, 30107 Murcia, Spain; dcasado9@ucam.edu (D.C.-G.); jreina@ucam.edu (J.R.-A.)

**Keywords:** anterior cruciate ligament reconstruction, conventional postoperative anterior cruciate ligament rehabilitation program, transcutaneous electrical nerve stimulation, percutaneous electrical nerve stimulation, long-term potentiation, quadriceps arthrogenic muscle inhibition

## Abstract

**Background/Objectives:** Quadriceps arthrogenic muscle inhibition (AMI) represents a key impairment following anterior cruciate ligament reconstruction (ACLR), contributing to quadriceps weakness. Although transcutaneous electrical nerve stimulation (TENS) and percutaneous electrical nerve stimulation (PENS) have been primarily investigated for analgesia, their effects on quadriceps strength in the early postoperative period remain underexplored. **Methods:** This study describes a single-blinded, parallel-group randomized controlled trial investigating the short-term effects of a single high-frequency TENS session and a novel long-term potentiation (LTP) PENS protocol on quadriceps strength and related clinical outcomes after ACLR. Fifty-four participants will be randomly allocated using block randomization in a 1:1:1 ratio to one of three groups: a control group (conventional post-ACLR rehabilitation only), a TENS group (conventional rehabilitation plus a single high-frequency TENS session), or a PENS group (conventional rehabilitation plus a single LTP PENS session). Participants will receive neuromodulatory intervention during the sixth postoperative week. The LTP PENS protocol consists of five 5 s stimulation bursts at 100 Hz and 250 μs pulse width and has only been investigated once in patients with upper limb pathology, underscoring its novelty in a postoperative setting. **Results:** The primary outcome is quadriceps maximal voluntary isometric contraction, selected as a clinically relevant surrogate of quadriceps activation deficits associated with AMI. Secondary outcomes include pain intensity, pressure pain threshold, knee range of motion, thigh muscle perimeter, knee effusion and swelling, and self-reported function and knee-related quality of life. Outcomes will be assessed at baseline, immediately post-treatment, and 1 and 7 days post-intervention by a blinded assessor.

## 1. Introduction

The anterior cruciate ligament (ACL) plays a critical role in knee stability, serving as the primary restraint against anterior tibial translation throughout the full range of knee flexion. ACL deficiency increases anterior subluxation of the tibiofemoral compartments under combined loads of anterior translation, internal rotation, and valgus stress, as observed in the pivot-shift phenomenon [1,2,3]. Moreover, the ACL contributes to controlling internal rotation, particularly near full extension [1,2,4].

ACL injuries are prevalent in the general population. Recent epidemiological data indicate that the annual incidence of ACL rupture is approximately 75 per 100,000 person-years in the general population of the United States, with higher rates observed among females (51.2%) and adolescents aged 10–19 years [5,6].

The American Academy of Orthopedic Surgeons recommends ACL reconstruction (ACLR) for patients with persistent instability, particularly young and physically active individuals, to restore knee stability, optimize joint function, and prevent secondary meniscal and cartilage damage [7]. Commonly used grafts include hamstring tendon autografts, bone–patellar tendon–bone autograft, quadriceps tendon autograft, and allografts. Autografts are generally favored in younger, active patients, whereas allografts are typically reserved for older or lower-demand populations [8,9,10].

Importantly, concomitant procedures to improve knee stability are increasingly performed. Since the description of the anterolateral ligament (ALL) in 2012, lateral extra-articular tenodesis (LET) has regained attention as an adjunct to ACLR to improve rotational stability [11,12,13,14,15]. Commonly applied techniques include the modified Lemaire and Arnold–Cocker procedures [13,16,17]. In the context of early quadriceps recovery, these procedures may influence neuromuscular activation patterns and postoperative rehabilitation; therefore, their potential impact is acknowledged in this trial.

Despite advances in surgical techniques and postoperative management, ACL re-rupture rates remain clinically relevant. Reported rates vary according to age, graft type, surgical technique, and activity level, ranging from 3.9% to 9% in the general population over 3–5 years, and reaching up to 19.7% among high-level athletes undergoing hamstring tendon reconstruction [18,19,20].

Following ACLR, the graft undergoes osseointegration at the tunnel interface and intra-articular remodeling, commonly referred to as ligamentization [21,22]. Understanding the biological processes and timing of each phase of graft healing is essential to optimize rehabilitation protocols and identify activities that may place excessive strain on the graft [21,23].

Quadriceps arthrogenic muscle inhibition (AMI) is a key impairment following anterior cruciate ligament reconstruction (ACLR), leading to persistent quadriceps weakness, altered neuromuscular activation, motor control deficits, and biomechanical gait alterations [24,25,26]. AMI arises from both peripheral and central mechanisms. Peripherally, inflammation, pain, and joint effusion alter afferent input from joint mechanoreceptors, resulting in reflex inhibition of quadriceps alpha motor neurons. Centrally, decreased corticospinal excitability and cortical plasticity changes reduce neural drive to the quadriceps, perpetuating impaired muscle activation [27,28].

These alterations lead to changes in muscle activation patterns and reduced motor unit recruitment, resulting in decreased quadriceps maximal voluntary contraction. Consequently, effective management of AMI during rehabilitation is essential to optimize early quadriceps activation [24,25,27]. The underlying peripheral and central mechanisms provide a rationale for targeted neuromodulatory interventions that may facilitate the restoration of quadriceps activation and strength.

Transcutaneous and percutaneous electrical nerve stimulation (TENS and PENS, respectively) have been predominantly investigated for analgesic effects [29,30,31,32]. However, evidence regarding their effectiveness on muscle strength remains limited. As far as we are aware, clinical trials investigating primarily pain outcomes with PENS are more numerous [33,34,35,36,37,38,39,40] than those focusing on quadriceps-specific strength outcomes [41], with only one study including a postoperative patient [40].

Although AMI is fundamentally a neural activation deficit, quadriceps maximal voluntary isometric contraction (QMVIC) was selected as the primary outcome because it represents a clinically relevant surrogate of the functional consequences of impaired quadriceps activation [42,43]. Accordingly, this study adopts a clinical-functional perspective, hypothesizing that TENS and PENS exert their effects primarily through direct neuromodulatory influences on muscle activation rather than solely via pain reduction.

A single-session intervention was deliberately chosen to assess the ability of neuromodulatory techniques to acutely enhance quadriceps activation during a critical postoperative period characterized by persistent AMI. The sixth postoperative week represents a clinically relevant time point: biological graft healing following the early healing phase permits safe neuromuscular loading, while neuromuscular inhibition persists despite conventional post-ACLR rehabilitation program (CRP) [21]. High-frequency TENS and a long-term potentiation (LTP) PENS protocol were selected for their capacity to induce rapid changes in neural excitability [44], enabling evaluation of whether even a single session can produce clinically meaningful improvements when combined with CRP. Moreover, to our knowledge, the administered LTP PENS protocol has previously been investigated only once in patients with arm pathology, underscoring its novelty in a postoperative context [45].

Accordingly, the primary objective of this randomized controlled trial is to determine whether adding a single session of high-frequency TENS or the novel LTP PENS protocol to a conventional post-ACLR rehabilitation program improves quadriceps maximal voluntary isometric contraction compared with rehabilitation alone.

Secondary objectives include evaluating the effects of these interventions on key clinical outcomes, including pain intensity, pressure pain threshold, knee range of motion, thigh muscle perimeter, intra-articular knee effusion, extra-articular knee swelling, and self-reported function and knee-related quality of life.

Exploratory analyses will assess whether these effects vary according to graft type or concomitant surgical procedures, contributing to a more precise understanding of how neuromodulatory rehabilitation strategies can be tailored to specific surgical approaches and patient characteristics. Aligning rehabilitation with surgical technique may inform more individualized, efficient, and evidence-based postoperative management.

## 2. Materials and Methods

The current study protocol follows the Standard Protocol Items: Recommendations for International Trials (SPIRIT) [46].

### 2.1. Study Design and Setting

A single-blinded randomized controlled trial will be conducted at Clínica CEMTRO (FIFA Medical Centre of Excellence), Madrid, Spain, in accordance with Consolidated Standards of Reporting Trials (CONSORT) guidelines [47,48]. The intervention and follow-up period for participants will be 7 days.

### 2.2. Ethics Approval and Enrollment of Participants

The study will be conducted in accordance with the Declaration of Helsinki [49,50] and the Spanish Law on Personal Data Protection and Guarantee of Digital Rights [51]. The protocol was approved by the Medical Direction for Education and Research and the Directorate of the Physiotherapy Department of Clínica CEMTRO, and subsequently by the Institutional Review Board (or Ethics Committee) of Universidad Católica San Antonio de Murcia (Protocol Code: CE112417). The trial was registered in the ClinicalTrials.gov database (identifier NCT06910150).

Patients beginning postoperative rehabilitation after anterior cruciate ligament reconstruction (ACLR) at Clínica CEMTRO will be invited to participate. Those who agree will sign an initial informed consent form authorizing the principal investigator to access their medical records and contact them directly. During the initial clinical assessment, participants will receive detailed information about the study and have the opportunity to ask questions. Volunteers meeting the inclusion criteria will then provide a second informed consent form and will receive a withdrawal form, with verbal reminders that they may revoke consent at any time without justification. Participants’ autonomy and data confidentiality will be ensured throughout the study.

### 2.3. Sample Size Calculation

Sample size was calculated using G*Power software (version 3.1) [52] for a repeated-measures analysis of variance with three groups. Assuming a medium effect size (f = 0.25), a power of 80% (β = 0.20), and a two-sided significance level of α = 0.05, a total sample size of 45 participants was required. The calculation was based on a correlation among repeated measures of 0.5 and a nonsphericity correction factor (ε) of 1. To account for an anticipated dropout rate of 20%, a total of 54 participants will be recruited, with 18 allocated to each group (control, TENS, and PENS).

### 2.4. Participants and Eligibility Criteria

Eligible patients will be aged 18–55 years old and will have undergone ACLR within the previous six weeks using hamstring tendon (HT), patellar tendon (BPTB), quadriceps tendon autografts, or allografts at Clínica CEMTRO. ACLR may be accompanied by concomitant surgical procedures on one or both menisci (repair, debridement, rasping, partial meniscectomy) and/or a Lemaire lateral extra-articular tenodesis. All patients will provide written informed consent.

Exclusion criteria will include: (1) contraindications for invasive procedures [53]; (2) contraindications for dynamometric assessment [41]; (3) history of lumbar hernia or protrusion; (4) receipt of alternative treatments outside the study (e.g., analgesics); (5) neuropathic pain in the lower limbs; (6) central nervous system disorders [39]; (7) history of neurological disorders or spinal surgery; (8) inability to reliably complete self-reported questionnaires [54]; (9) Personal Psychological Apprehension Scale score > 37.5 [55]; (10) absence from scheduled measurement sessions; and (11) voluntary withdrawal from the study.

The Personal Psychological Apprehension Scale (PPAS) is a valid and reliable instrument that provides a useful tool for assessing participants’ subjective perceptions. The PPAS consists of 15 items, each with four response options: (1) very rarely, almost never; (2) occasionally, hardly ever; (3) frequently, quite often; and (4) continuously, almost always. Total scores range from a minimum of 15 points to a maximum of 60 points [55].

This criterion is applied to ensure participant safety and compliance, because individuals with high psychological apprehension may experience discomfort or adverse reactions during the intervention. Notably, the same PPAS-based exclusion criterion has been applied in previous studies involving invasive physiotherapy interventions [56,57].

### 2.5. Allocation and Randomization

The randomization sequence will be generated by an independent statistician not involved in participant recruitment or outcome assessment. Randomization will be conducted prior to study initiation using Research Randomizer v4.0 [58]. Block randomization with a block size of three will ensure an equal number of participants across the three study groups [59]. A total of 54 participants will be allocated in 18 blocks of three, with each block containing one assignment to each group (1 = control, 2 = TENS, 3 = PENS), without repetition or predetermined order.

Stratified randomization by graft type or concomitant procedures was not implemented; participants were randomized without stratification to maintain study feasibility and integrity.

To ensure allocation concealment, the generated randomization sequence will be implemented using individual, sequentially numbered index cards indicating the assigned group. The cards will be placed in sealed opaque envelopes, which will be opened only after baseline assessments are completed by an independent assessor. This approach prevents recruitment and enrollment personnel from predicting future allocations, even with block randomization of size three. By combining independent sequence generation with secure envelope implementation, selection bias is minimized while ensuring correct assignment of the intervention.

### 2.6. Blinding

Due to the study design, the treating clinician cannot be blinded to group allocation. However, the outcome assessor will remain blinded to group allocation throughout the study period to maintain objectivity in data collection and analysis.

To minimize the risk of assessor unblinding, the assessor will not be present in the clinical setting during intervention delivery, and the clinician will remind participants not to disclose their assigned intervention during outcome assessments. Although the invasive nature of PENS may pose a theoretical risk, these procedures are expected to maintain assessor blinding throughout data collection.

To formally evaluate blinding integrity, the assessor will be asked at each outcome assessment (A2: immediately post-intervention; A3: 24 h post-intervention; and A4: 7 days post-intervention) to guess each participant’s group allocation and provide a confidence in this judgment. This procedure will allow monitoring of blinding effectiveness, particularly given that participant and treating clinician blinding is not feasible.

### 2.7. Interventions

The trial will comprise three groups: the control group (conventional post-ACLR rehabilitation program (CRP) only), the TENS group (CRP plus a single high-frequency TENS session), and the PENS group (CRP plus a single LTP PENS session).

The primary objective of this study is to evaluate the added efficacy of TENS or PENS when combined with CRP, rather than comparing these interventions against a placebo. The implementation of a sham intervention for TENS or PENS is inherently challenging because the sensory characteristics of both procedures make it difficult to create a fully convincing placebo. For TENS, the perception of electrical stimulation is readily apparent to participants, while for PENS, no sham needles are available.

To minimize the potential influence of performance and placebo-related effects, se-veral strategies will be implemented. Outcome assessors will remain blinded to group allocation. All participants will receive standardized instructions and procedures. Participants will be instructed not to reveal their assigned treatment to the assessor. The same CRP will be applied consistently across all groups. These measures aim to reduce expectancy bias and its impact on objective outcomes, such as pressure pain threshold and QMVIC.

**Figure 1:**
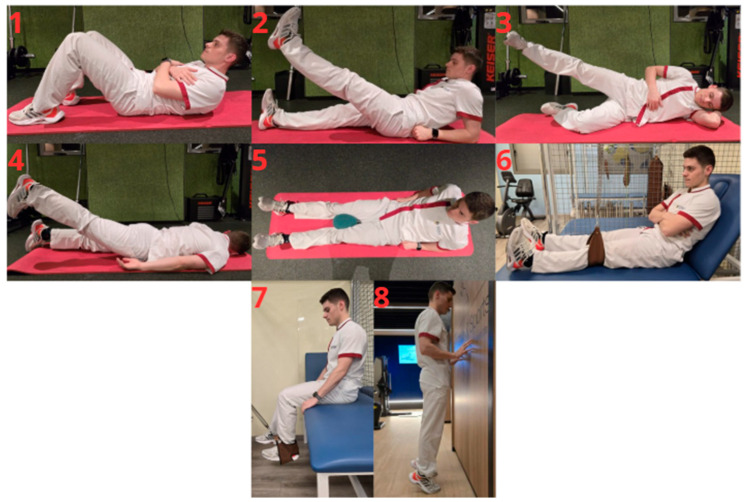
Photographic examples of the therapeutic exercises included in the conventional rehabilitation program (CRP): 1: Abdominal crunches; 2: Supine straight leg raises; 3: Side-lying straight leg raises; 4: Prone straight leg raises; 5: Inner thigh squeezes; 6: Isometric knee extension; 7: Isometric seated hamstring curl; 8: Calf raises.

All participants will perform CRP according to the graft ligamentization phase [21]. CRP will include passive knee mobilization (30 min) using a continuous passive motion device (Artromot^®^-K1, Chattanooga, Barcelona, Spain), manual therapy (≤15 min), and therapeutic exercise. Manual therapy will consist of passive knee joint mobilizations, drainage of the suprapatellar recess, surgical scar massage and myofascial soft tissue techniques. Therapeutic exercise will consist of 3 sets of 10 repetitions with 1 min rest intervals and will include the following exercises: abdominal crunches, supine, prone, and side-lying straight leg raises, inner thigh squeezes, isometric knee extensions, isometric seated hamstring curls, and calf raises [23]. Photographic examples of these exercises are provided in Figure 1.

Participants will perform the exercises at the same workload used prior to study initiation. The rehabilitation program was standardized and identical for all participants across groups, with no additional progression or load modification during the study.

All interventions will be delivered by the same experienced clinician specialized in invasive physiotherapy techniques. The Duo Tens Globus^®^ electrostimulator (Fisiomundo Tecnología Médica y Rehabilitación SL, Alicante, Spain) will be used to deliver a biphasic, symmetrical, square waveform current to the targeted motor points [45,60].

Once participants provide written informed consent, the blinded assessor will perform the baseline assessment (A1). Immediately afterward, the assessor will leave the clinical setting, and the clinician, if applicable, will administer a single 30 min TENS session or a single 5 min LTP PENS session. The clinician will also instruct all participants on the guidelines for performing the CRP over the following 7 days, which will constitute the total duration of the program. Table 1 summarizes the key characteristics of the interventions applied in each study group, highlighting the differences between the control group, the TENS group, and the PENS group.

Control Group: Participants will perform CRP only.

TENS Group: During the sixth postoperative week, participants will perform CRP and receive a single high-frequency TENS session. Two surface electrodes (5 × 5 cm) will be placed over the distal zone of the middle third of the vastus medialis and distal zone of the proximal third of the vastus lateralis [61,62]. Stimulation parameters will be 100 Hz frequency, 200 μs pulse width, intensity adjusted to each participants’ pain threshold, and duration 30 min. Participants will be allowed to adjust intensity during the session.

The TENS protocol specifies a reproducible criterion for adjusting intensity by increasing pulse amplitude to achieve a strong but comfortable sensation [60], tailored to each participant’s tolerance threshold and consistent with the scientific literature [30,32,63,64]. Moreover, evidence suggests that higher intensities produce greater effects and may prevent analgesic tolerance [30,32,63,65].

No predefined intensity algorithm will be used; however, the clinician provides consistent instructions for all participants in the TENS group to adjust stimulation according to their individual pain threshold during administration, ensuring standardized delivery.

When the high-frequency TENS session begins, the clinician will progressively increase stimulation intensity until the participant reports a strong but comfortable sensation [60]. To ensure reproducibility and transparent reporting of the delivered dose, the final stimulation intensity will be recorded for each session. The device allows a maximum output of 100 mA, and a standardized patient-reported perceived intensity rating (0–10 scale) will be recorded at session end. Only the final intensity will be reported, as no other parameters are adjusted during application. Stimulation intensity data will be reported descriptively to characterize the delivered dose.

PENS Group: During the sixth postoperative week, participants will perform CRP and receive a single long-term potentiation PENS session. Motor points will be identified according to Page et al. [62] and the needle puncture will be performed following the guidelines described by Velázquez-Saornil et al. [66]. A sterile acupuncture needle (0.30 × 40 mm, Tony^®^, Calvo Izquierdo S.L., Requena, Spain) will be inserted into the vastus medialis motor point. The knee will be passively flexed to 30°, and the skin will be disinfected with chlorhexidine. If the needle contacts the femoral cortex, it will be slightly withdrawn to avoid periosteal stimulation [60,67,68]. An electrode (5 × 5 cm) will be placed on the anteromedial knee to cover the infrapatellar branch of the saphenous nerve and medial femoral cutaneous nerves [69,70]. Electrical stimulation will be delivered at 100 Hz, 250 μs pulse width, at a perceptible but not painful intensity (200 μA above detection threshold), in five 5 s bursts with 55 s rest intervals [45].

Hemostasis will be performed for 60 s after needle withdrawal [45].

No adverse effects are expected due to the anatomical location of the puncture and the minimally invasive nature of the procedure. The puncture will be performed cranial to the suprapatellar recess and lateral to the sartorius muscle, posing no risk of intra-articular infection and avoiding the saphenous nerve in Hunter’s canal [71,72,73]. The intervention will be delivered by a qualified physical therapist with expertise in invasive approaches. All procedures will be conducted in the clinical setting of the Clínica CEMTRO, following standardized procedures [62,66].

In the unlikely event of an unexpected adverse reaction, the invasive intervention will be halted, and the adverse-event (AE) monitoring plan will be implemented, which will vary depending on the specific AE. Expected AEs for PENS include bleeding/bruising, neuropathic symptoms, vasovagal responses and infection signs.

The clinician delivering the intervention will collect all AE data immediately post-needling (A2) and at each follow-up assessment (24 h and 7 days post-intervention, A3 and A4, respectively). All AEs will be documented using a standardized form noting severity (mild/moderate/severe). Mild or moderate events will be managed in the clinical setting, while severe or unexpected events will be referred to hospital services for appropriate care.

As only non-anticoagulated participants are recruited [53], hemostasis for 60 s after needle withdrawal will address bleeding and bruising. If bleeding persists beyond 60 s, hemostasis will be continued until complete cessation. If the participant reports minimal neuropathic symptoms, the clinician will adjust the needle angulation until symptoms resolve. If this is insufficient, the clinician will repeat the puncture procedure.

In the presence of objective signs or subjective reports of a vasovagal response, the participant will be placed in a fully supine position. Legs will be elevated to promote venous return and to help maintain stable cerebral perfusion. If the participant reports a sensation of heat, the room environment will be adjusted by opening a window to reduce temperature and improve oxygen availability.

Although strict aseptic techniques will be applied as a preventive measure for infection, participants will be referred to hospital services for appropriate care should any signs of infection occur.

### 2.8. Outcomes

Assessments will be conducted at four time points: pre-treatment (A1), immediately post-treatment (A2), 24 h post-treatment (A3), and 7 days post-treatment (A4). All assessments will be conducted by an assessor blinded to group allocation and will be performed in the same clinical setting at Clínica CEMTRO (Madrid, Spain), under standardized conditions. The order of outcome assessment will be identical whenever applicable. All outcomes will be assessed according to the schedule presented in Table 2.

Baseline descriptive variables will include sex, age, body mass, height, BMI, limb dominance, operated limb, graft type (hamstring tendon autografts, bone–patellar tendon–bone autografts, quadriceps tendon autografts or allografts), associated lateral extra-articular tenodesis, and meniscal procedures (none, repair, debridement, rasping, partial meniscectomy). Surgery-related sensory disturbances (none, anesthesia, hypoesthesia, hyperesthesia, paresthesia) will also be recorded.

#### 2.8.1. Primary Outcome

Quadriceps maximal voluntary isometric contraction (QMVIC) of the operated limb will be measured using a calibrated hand-held dynamometer (Lafayette Manual Muscle Tester, Lafayette Instruments, Lafayette, IN, USA), which has demonstrated excellent intra-rater reliability [ICC = 0.98; 95% CI, 0.98–0.99] [74]. All measurements will be performed by an assessor experienced in hand-held dynamometry in previous research studies.

Participants will be seated with an upright trunk, 90° hip flexion, and 75° knee flexion [75,76]—a position associated with minimal stress on the ACL graft [77]. Thigh stabilization will be applied to prevent compensatory movements, and participants will be instructed to hold the edge of the treatment table if needed [75,78]. To ensure measurement validity, knee angle will be set using a 360° universal manual goniometer prior to each QMVIC assessment, following the guidelines of Norkin [79]. The dynamometer will be aligned with the lateral femoral condyle, and resistance will be applied to the anterior tibia 2 cm proximal to the malleoli [41,80].

Three maximal contractions of 5 s each will be performed, with 60 s of rest between trials. Standardized verbal encouragement will be provided during each contraction. The highest absolute force value (N) will be recorded [75,76,78,81]. Torque values will not be normalized to body mass or lever arm.

#### 2.8.2. Secondary Outcomes

Secondary outcomes will include pain intensity, pressure pain threshold (PPT), knee range of motion (ROM), thigh muscle perimeter, intra-articular knee effusion, extra-articular knee swelling, and self-reported function and knee-related quality of life.

Pain will be measured using the numerical rating scale (NRS) [82], PPT will be measured at six predefined anatomical points using an analog pressure algometer [83] and ROM will be measured with a universal goniometer [33,79]. Thigh perimeter and knee effusion will be measured with a flexible non-elastic measuring tape [84]. Knee swelling will be assessed clinically [84]. Self-reported function knee-related quality of life will be evaluated using the Lysholm Knee Score [85] and ACL-QoL-Sp [86].

**Pain Intensity**

Pain intensity will be assessed using the NRS, ranging from 0 (no pain) to 10 (maximum imaginable pain), given the general consensus that it provides greater validity compared with visual analog scales and facial or verbal pain rating scales [63].

**Pressure Pain Threshold**

Pressure pain threshold will be measured using an analog pressure algometer (FPK20, Wagner Instruments, Greenwich, CT, USA), consisting of a manometer connected to a 1 cm^2^ rubber probe, with values expressed in kg/cm^2^ (0–10 kg/cm^2^). Pressure will be applied perpendicularly at a constant rate of approximately 1 kg/s until the participant reported the first sensation of pain. At that point, pressure application will be discontinued and the corresponding measurement value recorded [87].

All measurements will be performed by an assessor with previous experience in pressure algometry assessments in prior research studies. Prior to the assessment, participants completed two familiarization trials on the hand. Three measurements will be collected at each anatomical site, with a 30 s interval between trials to prevent temporal summation. The mean of the three values will be used for statistical analysis. If a difference greater than 1 kg/cm^2^ was observed between two consecutive measurements at the same site, the highest value was discarded and an additional measurement was performed [87].

Measurements will be obtained at 5 predefined anatomical points of the operated limb [64], assessed in the same order at each evaluation: vastus lateralis (10 cm lateral to the midpoint of the superior patellar border), quadriceps tendon (3 cm proximal to the superior patellar border), vastus medialis (3 cm medial to the midpoint of the superior patellar border), patellar tendon (midpoint between the inferior patellar pole and the tibial tuberosity), pes anserinus (3 cm medial to the tibial tuberosity), in addition to the ipsilateral lateral humeral epicondyle. The algometer will be the only point of contact with the participant to avoid sensory interference [88].

**Knee Range of Motion**

Active and passive knee flexion and extension ROM will be measured using a 360° universal manual goniometer. The fulcrum will be positioned over the lateral femoral condyle, the proximal arm aligned with the lateral midline of the femur toward the greater trochanter, and the distal arm aligned with the lateral midline of the fibula toward the lateral malleolus [79].

Special care will be taken to avoid eliciting excessive pain during ROM assessment, as this could negatively impact the participant and potentially influence subsequent measurements [33].

**Thigh Muscle Perimeter**

Thigh muscle perimeter will be measured 10 cm proximal to the superior border of the patella using a flexible non-elastic measuring tape [84]. The corresponding measurement will also be recorded on the contralateral limb.

**Intra-Articular Knee Effusion**

Intra-articular knee effusion will be assessed on the basis of bulge and dancing patella signs and reported as to a four-grade scale: 0 (no effusion), 1 (<25 cm^3^, palpable by smoothing out the joint capsule), 2 (25–60 cm^3^, visible effusion), and 3 (>60 cm^3^, tight capsule) [84].

**Extra-Articular Knee Swelling**

Extra-articular knee swelling will be assessed by measuring the knee perimeter from the center of the patella using a flexible non-elastic measuring tape [84]. The corresponding measurement will also be recorded on the contralateral limb.

**Self-reported Function and Quality of Life**

Self-reported function and knee-related quality of life will be evaluated using the Lysholm Knee Scoring Scale [85] and the Spanish version of the Anterior Cruciate Ligament-Quality of Life questionnaire (ACL-QoL-Sp) [86], respectively. Both questionnaires will be administered only at baseline (A1) and at the final follow-up (A4).

The Lysholm Knee Scoring Scale [85] is the most widely used instrument for self-reported functional evaluation following ACLR and assesses limping, use of walking aids, locking, instability, pain, swelling, stair climbing ability, and squatting or crouching ability during activities of daily living. With a maximum possible score of 100 points, scores are classified as: (1) Poor: <65; (2) Fair: 65–83; (3) Good: 84–94; and (4) Excellent: >95.

**Figure 2:**
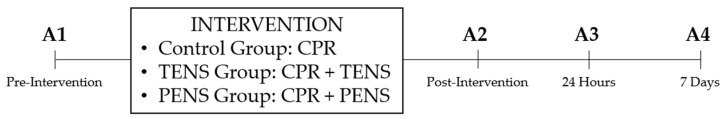
Experimental protocol timeline. CRP: conventional post-ACLR rehabilitation program; PENS: percutaneous electrical nerve stimulation; TENS: transcutaneous electrical nerve stimulation.

The study design and experimental protocol timeline are summarized in Figure 2.

### 2.9. Statistical Methods

All statistical analyses will be performed using SPSS statistical software for Windows (version 27.0; SPSS Inc., Chicago, IL, USA). The primary analysis will be conducted according to an intention-to-treat approach, using linear mixed-effects models, which accommodate repeated measures and handle missing data under the missing-at-random assumption.

A complementary per-protocol analysis will be conducted as a sensitivity analysis, including only participants who complete the assigned intervention and all scheduled assessments according to predefined criteria. This dual approach allows evaluation of the robustness of the primary findings.

Data normality will be assessed using the Shapiro–Wilk test. Descriptive statistics will be calculated for baseline characteristics, reporting means and standard deviations for normally distributed variables, and medians with interquartile ranges for non-normally distributed variables.

To evaluate intervention effects over time, repeated-measures analysis of variance (ANOVA) will be applied to outcomes meeting normality assumptions. The within-subject factor will be time (pre-treatment, immediate post-treatment, 24 h post-treatment, and 7 days post-treatment), and the between-subject factor will be group (control: CRP only; TENS: CRP plus a high-frequency TENS session; PENS: CRP plus a LTP PENS session). Bonferroni correction will be used for post hoc comparisons. Sphericity assumptions will be assessed, and Greenhouse–Geisser corrections will be applied as appropriate.

To provide a more flexible and robust analysis of repeated measures, linear mixed-effects models will additionally be applied. These models will include the fixed effects of group, time, and the group × time interaction. They allow inclusion of all available data and appropriately handle missing observations under the missing-at-random assumption without requiring complete cases or sphericity.

Exploratory multivariable analyses will be performed to investigate associations between clinical outcomes and sociodemographic and surgical-related variables, focusing on relationships with surgical procedures. Potential confounding effects of graft type and concomitant procedures on early quadriceps outcomes will not be formally tested, and any subgroup analyses will be limited to descriptive reporting.

A two-sided significance level of α = 0.05 will be adopted, and results will be reported with 95% confidence intervals.

## 3. Discussion

The present randomized controlled trial protocol has been designed based on previously published neuromodulatory interventions [45,60], assessment timing [45,89], and outcome measurement procedures [75,76,78,79,80,81,82,83,84,85,86]. These design choices ensure methodological rigor and clinical relevance. These methodological approaches will be applied to the early postoperative rehabilitation setting following ACLR.

Previous evidence has highlighted the importance of investigating the variability in the application of intramuscular electrical stimulation (IMES) in the vastus medialis in patients undergoing ACLR. These studies have proposed designs very similar to the current protocol [63,90]. Framing this trial as a complementary study to the existing literature emphasizes its potential contribution to refining clinical application and understanding of IMES in early postoperative rehabilitation, bridging gaps between experimental evidence and practical implementation.

### 3.1. Potential Impact and Significance of the Study

To the best of the authors’ knowledge, Caballero et al. [40] is the only study that has investigated the effectiveness of PENS in postoperative patients. This study protocol also involves participants undergoing ACLR. However, Caballero et al. [40] applied two sessions of a low-frequency (2 Hz, 240 μs pulse width) biphasic rectangular-pulse current targeting the femoral nerve in the peripheral nervous system.

Hsieh and Lee [89] also evaluated the effects of a single session of TENS and PENS (IMES). However, this approach differs from the present study protocol in that alternating frequencies (3–15 Hz) were delivered at acupuncture points for 15 min in patients with low back pain.

Similarly, Elbadawy [60] compared the effectiveness of high-frequency TENS and PENS in patients with advanced knee osteoarthritis. In that study, high-frequency TENS (100 Hz, 200 μs pulse width for 30 min) and high-frequency PENS (periosteal stimulation therapy using a symmetrical biphasic modified square wave at 100 Hz for 30 min) were applied once weekly for 10 weeks, in addition to a home exercise program prescribed to both groups.

In contrast to the previously cited studies [40,60,89], Álvarez-Prats et al. [41] specifically examined the effects of PENS on QMVIC. However, their approach [41] differs from the present protocol in that a single session of a biphasic asymmetric electrical current (10 Hz, 240 μs pulse width) targeting the femoral nerve in the peripheral nervous system was administered in non-postoperative participants.

These studies highlight the potential neuromodulatory benefits of TENS and PENS, yet evidence in the early postoperative ACLR population remains scarce. Despite advances in ACLR techniques, postoperative functional deficits remain significant challenges, often limiting early rehabilitation progress.

The present study hypothesizes that the addition of a single session of TENS or PENS to a CRP, based on manual therapy and therapeutic exercise, may produce short-term improvements in key clinical outcomes during the early postoperative period. If effective, such single-session applications could represent a potentially time- and cost-efficient adjunct, allowing evaluation of acute effects without implying sustained or long-term clinical benefits.

Moreover, most existing literature on TENS and PENS has been conducted in non-postoperative populations, with a predominant focus on pain outcomes rather than muscle strength. In the present trial, QMVIC was selected as the primary outcome as a clinically relevant performance-based surrogate of neuromuscular activation deficits associated with AMI, reflecting functional recovery priorities in post-ACLR patients.

It should be noted that maximal voluntary isometric strength reflects the effective recruitment of the muscle motor unit. Quadriceps AMI can markedly reduce maximal voluntary isometric strength, which is also correlated with maximal concentric and eccentric strength. Consequently, while QMVIC does not directly isolate neural mechanisms, it captures the integrated functional manifestation of impaired quadriceps activation and serves as a relevant outcome in neuromuscular assessments [41].

It is acknowledged that QMVIC in the early postoperative stage may be influenced by pain, knee effusion, and motivational factors; however, this multidimensional sensitivity also reflects the clinical reality of early rehabilitation, where neuromuscular inhibition, symptoms, and voluntary drive interact to constrain quadriceps force production.

### 3.2. Limitations

It should be acknowledged that this randomized controlled trial protocol presents some limitations that should be considered when interpreting future findings.

First, the lack of access to ultrasound guidance made it impossible to apply PENS at the femoral nerve level following previously published approaches [40,41]. Instead, PENS will be applied to the motor point of the vastus medialis, which is a plausible target due to its relatively high innervation density, clustering a large number of motor endplates, and may enhance muscle responsiveness [91,92]. Nevertheless, evidence supports that intramuscular electrical stimulation (IMES) is effective in reducing pain intensity [34], pressure pain threshold [93], and improving functional performance [93] and range of motion [94].

While ultrasound guidance was not employed, the clinician will perform a systematic approach based on established anatomical references. This approach will maximize the consistency of motor-point targeting across all group PENS participants [61,62]. With the participant in the supine position, the inguinal ligament point traversed by the femoral nerve (FNIL) was identified, which is located at an average distance of 6.44 cm (6.44 ± 1.03 cm) from the anterior superior iliac spine. Next, the distance from the FNIL to the patella was measured with a flexible non-elastic measuring tape for each participant (mean 40.19 ± 2.11 cm). This distance was multiplied by the ratio of FNIL to the motor point of the vastus medialis, which is 0.55 (mean 22.19 ± 3.04 cm). The resulting distance was marked on the participant’s vastus medialis motor point to guide the subsequent needling procedure. To ensure reliability, all measurements and anatomical landmarks of the systematic approach were performed by the same experienced clinician.

Second, patient blinding is not feasible, as the superficial TENS intervention is clearly distinguishable from the invasive PENS procedure. However, several measures are implemented to minimize the risk of assessor unblinding. The outcome assessor will not be present in the clinical setting during intervention delivery, and the treating clinician will remind participants not to disclose their assigned intervention during outcome assessments. Although the invasive nature of PENS may pose a theoretical risk of unblinding, these procedures are expected to preserve assessor blinding throughout data collection, thereby minimizing measurement bias.

Third, this study focuses on short-term outcomes assessed up to seven days post-intervention, which aligns with typical follow-up durations reported in the literature for PENS interventions [34,35,40]. While this design is suitable for evaluating early neuromuscular effects in the postoperative phase, it does not allow conclusions regarding the persistence of these effects or their influence on longer-term functional recovery following ACLR.

Finally, variability in individual response to neuromodulatory interventions, as well as potential heterogeneity in graft type and associated surgical procedures (meniscal procedures or lateral extra-articular reinforcement), may influence short-term outcomes. These factors should be considered when interpreting the findings and planning future studies.

### 3.3. Contributions to Postoperative Rehabilitation

This randomized controlled trial is expected to provide preliminary evidence on the short-term effects of a single session of TENS and PENS on key clinical outcomes during the early postoperative phase following ACLR. The findings may help inform whether single-session neuromodulatory interventions could reduce immediate treatment burden, support early functional recovery, and explore whether the short-term effects of TENS or PENS are more effective in certain graft types or associated surgical procedures (e.g., meniscal procedures or lateral extra-articular reinforcement). All interpretations are limited to short-term outcomes, and further studies with longer follow-up are required to determine the persistence and clinical relevance of these effects.

These exploratory multivariable analyses investigating whether the short-term effects of TENS or PENS differ according to graft type or associated surgical procedures (e.g., meniscal procedures or lateral extra-articular reinforcement) are primarily hypothesis-generating. The conclusions of these exploratory multivariable analyses are likely underpowered, and any observed trends should not be interpreted as definitive evidence.

Furthermore, this study could open new scientific pathways for the application of TENS and PENS protocols in other musculoskeletal or post-traumatic populations, and potentially inform the development of novel interventions based on the same underlying physiological principles [44].

To the best of the authors’ knowledge, the specific LTP protocol applied has only been investigated once in the peripheral nervous system of patients with upper limb pathology [45]. This protocol is characterized by the application of a biphasic, symmetrical, square-wave current at 100 Hz with a 250 μs pulse width, delivered at a perceptible but non-painful intensity in five 5 s bursts with 55 s rest intervals. Its novelty lies in being applied for the first time as intramuscular electrical stimulation in an early post-ACLR context.

## 4. Conclusions

This randomized controlled trial is designed to determine whether the addition of a single session of high-frequency TENS or a novel long-term potentiation (LTP) PENS protocol to a conventional post-ACLR rehabilitation program, which includes manual therapy and therapeutic exercise, provides additional short-term benefits compared with conventional rehabilitation alone in patients undergoing ACLR. The primary outcome will assess quadriceps maximal voluntary isometric contraction. Secondary outcomes will include pain intensity, pressure pain threshold, knee range of motion, thigh muscle perimeter, knee effusion and swelling, and self-reported function and knee-related quality of life.

Exploratory multivariable analyses will investigate whether the effects of these interventions differ according to graft type or concomitant surgical procedures. The findings from this study may help guide the tailoring of postoperative rehabilitation strategies to specific surgical approaches and patient profiles.

## Figures and Tables

**Table 1:** Comparative characteristics of the interventions applied in the three study groups.

	Control Group	TENS Group	PENS Group
Sample recruitment	Week 6 post-ACLR	Week 6 post-ACLR	Week 6 post-ACLR
CRP	Yes	Yes	Yes
Neuromodulatory intervention	None	HF-TENS	LTP PENS
Mode of application	-	Transcutaneous (surface electrodes)	Percutaneous (needle insertion)
Skin preparation	-	-	Chlorhexidine disinfected before needle insertion
Target tissue	-	Vastus medialis motor point	Vastus medialis motor point
		Vastus lateralis motor point	Infrapatellar branch of the saphenous nerve
Type of current	-	biphasic, symmetrical, square-wave	biphasic, symmetrical, square-wave
Frequency	-	100 Hz	100 Hz
Pulse width	-	200 μs	250 μs
Intensity	-	Adjusted to individual pain threshold	Perceptible but non-painful (≈200 μA above detection threshold)
Stimulation pattern	-	Continuous	Five 5 s bursts with 55 s rest intervals
Session duration	-	30 min	5 min
Number of sessions	-	Single session	Single session
Blinding assessment	Same assessor	Same assessor	Same assessor
Clinician delivering intervention	-	Same experienced clinician	Same experienced clinician

ACLR: Anterior cruciate ligament reconstruction; CRP: Conventional post-ACLR rehabilitation program; HF-TENS: High-frequency transcutaneous electrical nerve stimulation; LTP PENS: Long-term potentiation percutaneous electrical nerve stimulation; μA: Microamperes.; μs: Microseconds.

**Table 2:** Schedule of outcome assessments at each time point.

	Pre-Intervention Assessment (A1)	Post-Intervention Assessment (A2)	24 h Follow-Up Assessment (A3)	7 Day Follow-Up Assessment (A4)
Descriptive variables	X			
QMVIC	X	X	X	X
Pain intensity	X	X	X	X
PPT	X	X	X	X
ROM	X	X	X	X
Thigh muscle perimeter	X	X	X	X
Knee effusion	X	X	X	X
Knee swelling	X	X	X	X
Functional performance	X			X
Quality of life	X			X

PPT: Pressure pain threshold; QMVIC: Quadriceps maximal voluntary isometric contraction; ROM: Range of motion.

## Data Availability

The data generated in this study will be included in the results of the published article.

## Abbreviations

The following abbreviations are used in this manuscript:

ACL	Anterior Cruciate Ligament
ACLR	Anterior Cruciate Ligament Reconstruction
AMI	Arthrogenic muscle inhibition
CRP	Conventional Post-ACLR Rehabilitation Program
LTP	Long-Term Potentiation
QMVIC	Quadriceps Maximal Voluntary Isometric Contraction
TENS	Transcutaneous Electric Nerve Stimulation
PENS	Percutaneous Electric Nerve Stimulation
